# Intestinal dysbiosis in children with short bowel syndrome is associated with impaired outcome

**DOI:** 10.1186/s40168-015-0084-7

**Published:** 2015-05-04

**Authors:** Helene Engstrand Lilja, Hugo Wefer, Niklas Nyström, Yigael Finkel, Lars Engstrand

**Affiliations:** Department of Women’s and Children’s Health, Uppsala University, Uppsala, 751 85 Sweden; Department of Microbiology, Tumor and Cell Biology and Science for Life Laboratory, Karolinska Institute, Stockholm, 171 77 Sweden; Department of Clinical Science and Education, Karolinska Institute, Stockholm, 118 83 Sweden; Sachs’ Children’s and Youth Hospital, Stockholm, 118 83 Sweden; Clinical Genomics Facility, Science for Life Laboratory, Solna, 171 65 Sweden

**Keywords:** Dysbiosis, Short bowel syndrome, Bacterial diversity, Gut microbiota

## Abstract

**Background:**

The composition of the intestinal microbiota seems to be an important factor in determining the clinical outcome in children with short bowel syndrome (SBS). Alterations in the microbiota may result in serious complications such as small bowel bacterial overgrowth (SBBO) and intestinal mucosal inflammation that lead to prolonged parenteral nutrition (PN) dependency with subsequently increased risk of liver failure and sepsis. To date, there are no reported mappings of the intestinal microbiome in children with SBS. Here, we present the first report on the intestinal microbial community profile in children with SBS.

**Findings:**

The study includes children diagnosed with SBS in the neonatal period. Healthy siblings served as controls. Fecal samples were collected, and microbial profiles were analyzed by using 16S rRNA gene sequencing on the Illumina MiSeq platform. We observed a pronounced microbial dysbiosis in children with SBS on PN treatment with an increased and totally dominating relative abundance of *Enterobacteriacae* in four out of five children compared to children with SBS weaned from PN and healthy siblings.

**Conclusions:**

The overall decreased bacterial diversity in children with SBS is consistent with intestinal microbiome mappings in inflammatory bowel diseases such as Crohn’s disease and necrotizing enterocolitis in preterm infants. Our findings indicate that intestinal dysbiosis in children with SBS is associated with prolonged PN dependency.

**Electronic supplementary material:**

The online version of this article (doi:10.1186/s40168-015-0084-7) contains supplementary material, which is available to authorized users.

## Findings

### Background

Pediatric intestinal failure (IF) has been defined as the inability of the gastrointestinal tract to sustain adequate growth, hydration, and electrolyte homeostasis in children without parenteral nutrition (PN). Short bowel syndrome (SBS) is the most common cause of pediatric IF. The condition is caused by massive small bowel resections due to necrotizing enterocolitis (NEC) or volvulus and congenital malformations such as gastroschisis and jejunal atresia. Neonatal SBS is a disease with a high morbidity and mortality [1]. The medical management of SBS aims to establish full enteral/oral feedings and weaning from PN.

The intestinal microbiota seems to be a major factor in determining the successful clinical outcome in SBS defined as independence of PN treatment and intestinal adaptation. Alterations in the microbiota can result in serious complications such as small bowel bacterial overgrowth (SBBO) and intestinal mucosal inflammation that may prevent weaning from PN by compromising intestinal absorptive functions [2,3]. Luminal/oral antibiotic long-term treatment has been recommended for SBBO in children [4,5]. Most cases of SBS occur in neonates during a period when the sterile intestines normally are colonized by bacteria, reaching a microbial profile characteristic of the adult gastrointestinal tract around 2 to 4 years of age [6]. A disruption in the balanced intestinal microbial community, that is, dysbiosis, with an increased relative abundance of facultative anaerobic *Enterobacteriaceae* in the large bowel is seen in inflammatory bowel disease (IBD) in mouse models, in humans with Crohn’s disease, and in NEC in preterm infants [7,8].

To date, there are no reported mappings of the intestinal microbiota in children with SBS. Here, we present the first report on the microbial profile in children with SBS by using 16S rRNA gene sequencing on the Illumina MiSeq platform.

### Methods

#### Patients

This study was approved by the regional committee on medical research ethics in Uppsala (Dnr2012/002). Informed written consent for sample collection and subsequent analyses was obtained from the parents. Characteristics of the study group and corresponding healthy siblings are demonstrated in Table 1. The study includes 11 children between the age of 1.5 to 7 years diagnosed with IF/SBS in the neonatal period, of whom two are from a set of triplets (2A and 3A) (Table 1). All children except one were prematurely born. Child 1A, 8A, and 13A underwent bowel lengthening procedure with serial transverse enteroplasty (STEP) [9]. Five children were not weaned from PN at the time of the study. Seven healthy siblings served as controls. Children on PN had an oral and/or enteral intake of lactose-free hydrolysed protein formula and an age-appropriate intake of solid foods with reduction in disaccharide content according to Table 1.

**Table 1:** **Characteristics of the study group and corresponding healthy siblings**

**Child**	**GA**	**Age (year)**	**Diagnosis**	**Remaining small bowel length (cm)**	**Remaining ICV**	**Remaining colon length**	**Proportion PN of daily nutritional intake**	**Shannon index**
1A^a^	35	4	Jejunal atresia	20 jejunum	No	1/3	75%	0.104
3A^a,b^	23	3	NEC	10 jejunum	No	1/2	70%	3.29
8A^a^	33	4	Gastroschisis	15 jejunum	No	1/2	30%	0.099
9A^a^	25	4	NEC	10 jejunum	No	1/2	80%	1.63
12A	41	1.5	Volvulus	27 jejunum	No	1/2	50%	1.16
2A^b^	23	3	NEC	All, (only 2 cm resection)	Yes	All	Off PN	4.67
4A	23	2.5	NEC	30 jejunum + 5 ileum	Yes	All	Off PN	4.21
11A	24	3	NEC	5 jejunum + 17 ileum	Yes	All	Off PN	4.30
13A	34	7	Jejunal atresia	35 jejunum	No	1/2	Off PN	3.36
16A	24	6	Volvulus	45 jejunum + 25 ileum	No	2/3	Off PN	3.38
18A	26	4	NEC	10 jejunum + 20 ileum	Yes	Yes	Off PN	3.17
*Healthy sibling*		*Age* (*years*)	*Shannon value*					
2C1		10	5.60					
2C2^b^		3	4.97					
11C1		11	6.12					
11C2		6	6.59					
12C		13	6.39					
13C1		11	6.56					
13C2		2	6.89					

^a^ = SBBO, ^b^ = triplets, GA = gestational age.

#### Data collection and statistical analysis

Fecal samples were collected and stored at −80°C until analysis. DNA was extracted from each fecal sample with ultra-clean fecal DNA isolation kit (MoBio, Naxo Ltd, Tartu, Estonia) according to the manufacturer’s instructions.

Sequencing libraries were prepared by amplifying the V3-V4 region of the 16S rRNA gene using the 341f-805r primers, described by Hugerth *et al*. [10]. After the initial amplification, a second PCR was performed to attach Illumina adapters as well as barcodes that allows for multiplexing. Samples were sequenced using the IlluminaMiSeq, producing in total 10,136,440 2 × 300 base pair reads with an average of 307,165 reads per sample. Primer sequences were trimmed away, and the paired-end reads produced by the sequencing instrument were merged using SeqPrep version 1.1 (https://github.com/jstjohn/SeqPrep) with default parameters and thereafter processed with the QIIME 1.8.0 pipeline (Quantitative Insight into Microbial Ecology) [11]. Merged reads were randomly subsampled to an even depth of 151,610 reads per sample, which was the minimum number of reads among the samples. Using the UCLUST [12] algorithm built into the QIIME pipeline, sequences were clustered at 97% identity against the Greengenes reference database producing 4,216 OTUs (operational taxonomic units) [11]. For each sample; number of non-singleton OTU as well as most dominant OTU, with respective description, is presented in the supplemental data (Additional file 1). Shannon indexes for diversity were calculated for SBS children on and off PN and tested for significance with Wilcoxon rank-sum test. Using the QIIME pipeline, unweighted UniFrac distances were produced and used for investigation of beta diversity through plotting PCA coordinates. Details on 16S rRNA gene primers, amplification conditions, and sample barcodes are shown in supplemental data (Additional file 2).

### Results

**Figure 1:**
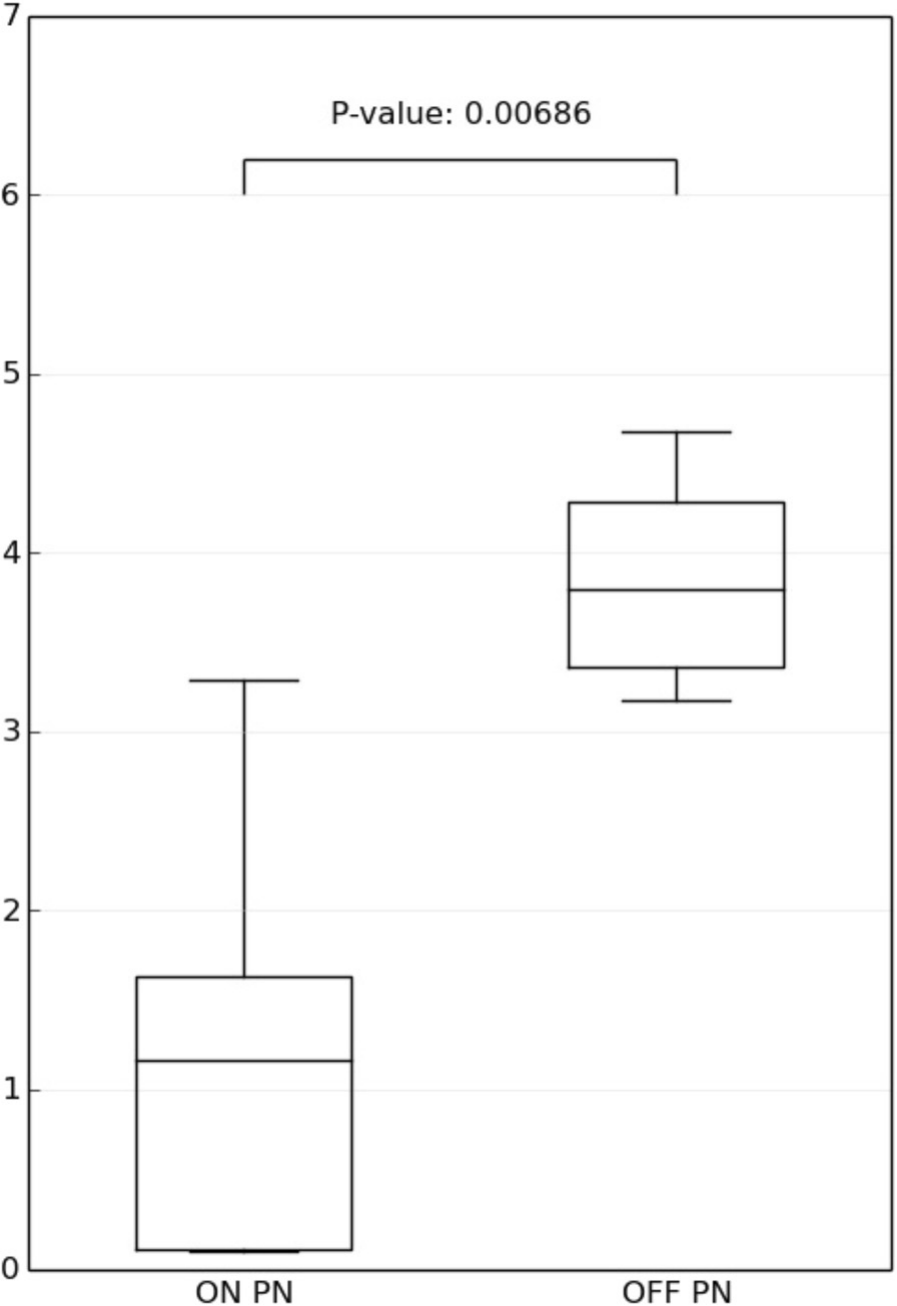
Shannon diversity index in children with SBS still on PN compared to children weaned from PN.

Figure 1 shows that Shannon diversity index is significantly reduced in children with SBS still on PN compared to children weaned from PN. None of the children on PN had remaining ICV.

**Figure 2:**
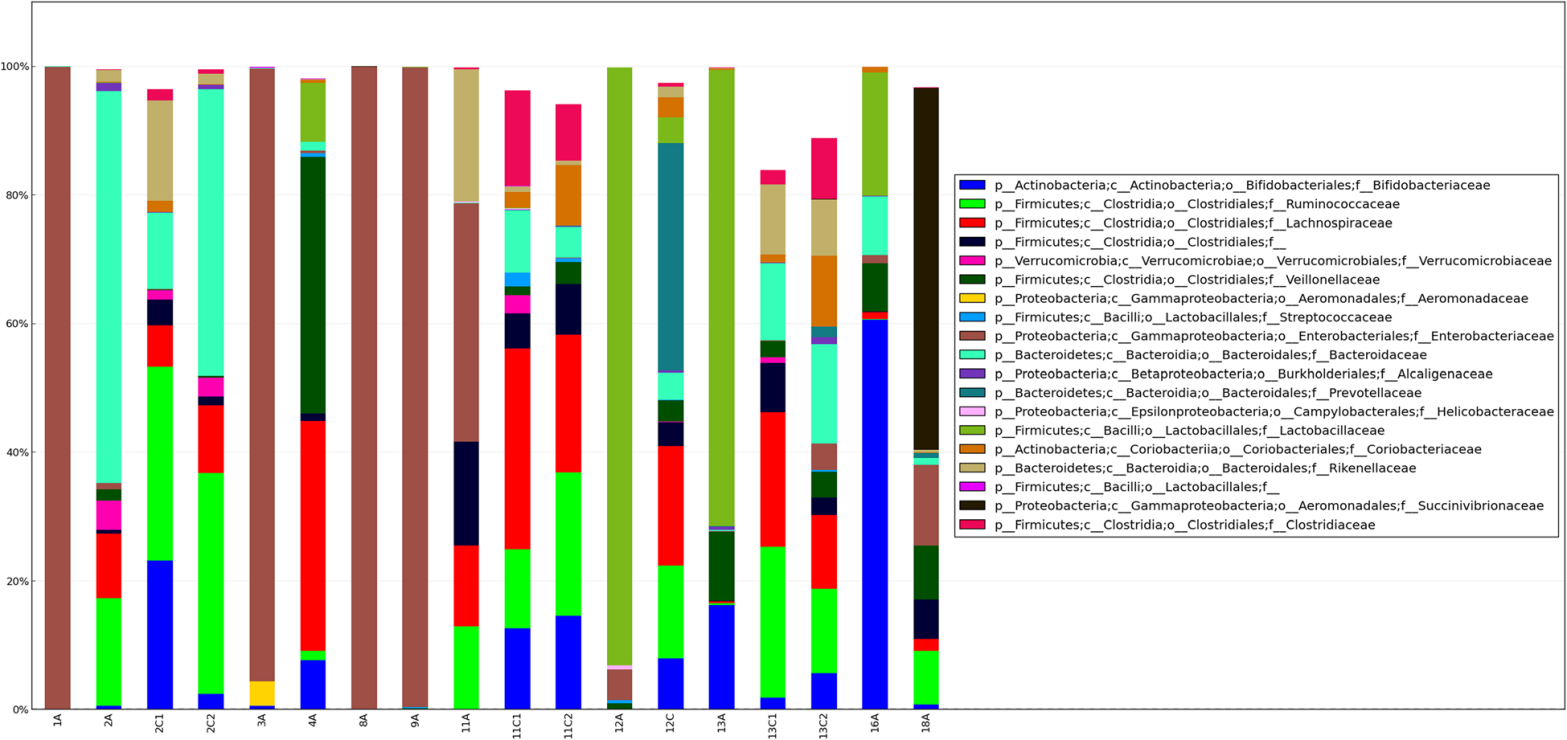
Microbial communities in children with SBS on PN (1A, 3A, 8A, 9A, 12A), SBS children weaned from PN (2A, 4A, 11A, 13A, 16A, 18A), and siblings (2C1, 2C2, 11C1, 11C2, 12C, 13C1, 13C2). The figure is showing the relative abundance of the 19 most common taxonomic families that accounts for at least 84% of the abundance in all samples.

In children still on PN, four out of five (1A, 3A, 8A, and 9A) were examined for several episodes of suspected SBBO, also at the time of fecal sampling (Table 1). They were treated with oral metronidazole, trimethoprim-sulfamethoxazole, gentamicin, or amoxicillin-clavulanic acid. In these patients, *Enterobacteriacae* was the most relative abundant taxonomic family and totally dominated the microbial community in these children (Figure 2). The remaining patient in this group (12A), still on PN and with a reduced Shannon diversity index, showed a relative abundant dominance of *Lactobacillaceae* followed by *Enterobacteriacae.* Altogether, a high relative abundance of *Enterobacteriacae* was associated with SBS in 6 out of 11 patients (1A, 3A, 8A, 9A, 11A, and 12A). In the remaining five SBS patients, all off PN (2A, 4A, 13A, 16A, and 18A), there was a more diverse microbiota composition and a more uniform distribution of taxonomic families. However, none except one (2A) of the SBS children reached Shannon diversity indexes at the same levels as the controls (Table 1). In one of the children still on PN (1A), upper and lower endoscopy with biopsies revealed macroscopic and histopathologic acute inflammation in the stomach, duodenum, small bowel, and proximal colon. In child 3A, also on PN, upper endoscopy with biopsies demonstrated small bowel villous atrophy.

**Figure 3:**
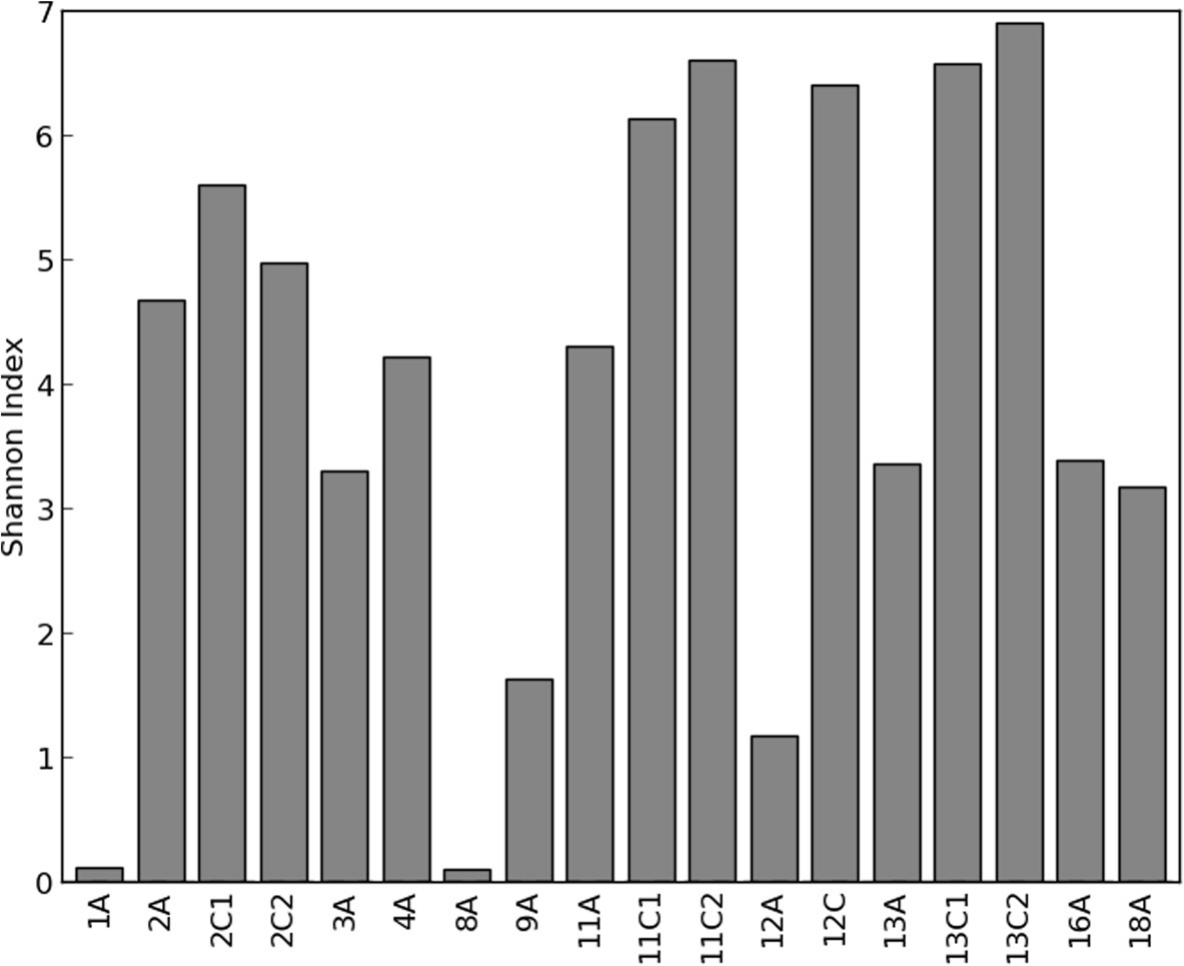
Shannon diversity index in children with SBS on PN (1A, 3A, 8A, 9A, 12A), SBS children weaned from PN (2A, 4A, 11A, 13A, 16A, 18A), and siblings (2C1, 2C2, 11C1, 11C2, 12C, 13C1, 13C2).

**Figure 4:**
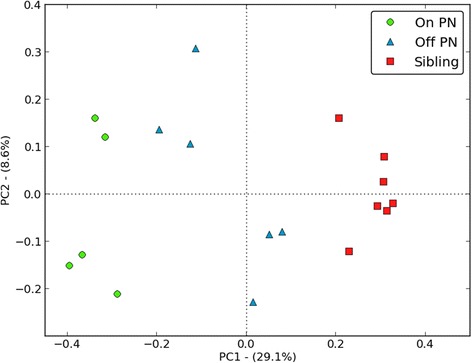
PCoA plot describing unweighted UniFrac distance between samples. Pairwise distances between all samples are projected onto a two-dimensional space where the axis PC1 describes the highest degree of variation. Samples that are clustered closely together are thus considered to share a larger proportion of the phylogenetic tree in comparison to samples that are more separated.

In Figure 3, the Shannon diversity indexes and, in Figure 4, the unweighted UniFrac distances in children with SBS on PN (1A, 3A, 8A, 9A, 12A), SBS children weaned from PN (2A, 4A, 11A, 13A, 16A, 18A), and siblings (2C1, 2C2, 11C1, 11C2, 12C, 13C1, 13C2) are compared.

We had the unique opportunity to study triplets representing all three groups. Child 2A and child 3A were male triplets born at 23 weeks of gestation. Both boys suffered from NEC in the neonatal period resulting in small bowel resections (Table 1). The third triplet boy (2C2) remained healthy. In child 2A, only 2 cm of the small bowel was resected, however he developed IF after extensive NEC and became dependent on PN. During PN treatment, he had no signs of SBBO. He was weaned to full oral feeding and without antibiotics 3 months before the time of fecal sampling. His intestinal bacterial diversity was similar to his healthy brother (SDI 4, 67 and 4, 97, respectively).

Child 12A was treated with antibiotics only during the first 2 weeks postnatally, had no signs of SBBO, and weaning from PN advanced but slowly. Her fecal bacterial diversity showed *Lactobacillacae* as the most relative abundant taxonomic family in accordance to our previous findings (Figure 2) [13]. We could detect *Clostridium difficile* in two out of ten SBS patients (patient 2A and 11A) and in very low relative abundance (data not shown).

### Conclusions

The tendency for SBBO and bowel inflammation to delay or prevent weaning from PN in these children with SBS seems to be related to microbial dysbiosis in the intestinal tract. This finding is in accordance with a previous study demonstrating that PN administration was independently associated with SBBO [14]. Influence of PN on the change in profile due to starvation of the microbiome is unlikely since malabsorption of oral/enteral nutrition is the major problem in SBS. In general, the observed changes in the microbiota in SBS children are most likely both at cause and a consequence of the disease status of the child. The limitation of the study is the small study group, and confounding factors that might influence the results are age, intestinal length, and antibiotic treatment. However, the cohort in the present study represents children with SBS in the clinical practice.

In our center, we treat SBBO with oral antibiotics as recommended by other centers [4-6]. However, it is most likely that antibiotics will further contribute to dysbiosis in these children. In children with SBS, normal colonization is disrupted due to early and frequent antibiotic use and decreased bacterial diversity allows potential pathogenic bacteria to expand. Antibiotics have been suggested to lower colonization resistance against *Enterobacteriacae* such as *Escherichia coli* and *Salmonella enterica*, by increasing the inflammatory tone of the intestinal mucosa [15]. Most frequently, probiotics are used to modify the intestinal microbiota in SBS; however, there are conflicting findings and reports of probiotic-associated septicemia [16].

The overall decreased bacterial diversity in our children with SBS is consistent with intestinal dysbiosis in IBD patients, infants with NEC, and has also been described in a piglet model of SBS [7,8,17,18]. In addition, children with recurrent *C. difficile-*associated diarrhea show a decreased fecal diversity with a reduction of *Bacteriodetes* and *Firmicutes* [19]. In these children, fecal microbiota transplantation (FMT) has a success rate greater than 90%. Such treatment has also been successfully used as a complement to treat IBD [19,20]. Consequently, FMT could prove to be a treatment alternative in carefully selected cases of SBS with dysbiosis. However, since children with SBS often are vulnerable due to their initial health status, the difficulties and risks of FMT must be considered. Although the incidence of severe side-effects is rare, one such risk is contracting illness from the donor where asymptomatic microorganisms that cause no problems in a healthy donor may cause a reaction in the recipient. In addition, mass arrival of a new microbiota may also trigger autoimmune illness as well as bacteria and septic shock. Extra care should also be taken with FMT if the patient has any sign of immunodeficiency [21].

This is the first report describing the intestinal microbial profile in children with SBS using next-generation sequencing. We observed a pronounced microbial dysbiosis in children with SBS still on PN compared to children weaned from PN with an increased relative abundance of proteobacteria, most of whom has been long-term treated with antibiotics. Our findings indicate that intestinal dysbiosis in children with SBS is associated with impaired outcome with prolonged PN dependency. Future studies need to find out new strategies to treat intestinal dysbiosis in these children.

## Availability of supporting data

The data sets supporting the results of this report are included within the article (and its additional file). The sequence data sets are available in the SRA repository, http://www.ncbi.nlm.nih.gov/bioproject/275923.

## Additional files

Additional file 1: Table S1. (excel) SBS OTU information: for each sample; number of non-singleton OTU as well as most dominant OTU, with respective description is presented. Additional file 2: Table S2. Sequence and index data: summary of 16S rRNA gene primers, amplification conditions and sample barcodes.

## Abbreviations

FMT: Fecal microbiota transplantation
IBD: Inflammatory bowel disease
ICV: Ileocecal valve
IF: Intestinal failure
PN: Parenteral nutrition
SBBO: Small bowel bacterial overgrowth
SBS: Short bowel syndrome
