# Supplementary material for: Intestinal dysbiosis in children with short bowel syndrome is associated with impaired outcome
Source: Microbiome. 2015 May 4;3:18. doi: 10.1186/s40168-015-0084-7 (PMC4418071; doi:10.1186/s40168-015-0084-7)
Supplement: Additional file 2: Table S2. — Sequence and index data: summary of 16S rRNA gene primers, amplification conditions and sample barcodes. [file 40168_2015_84_MOESM2_ESM.pdf]

341F primer: CCTACGGGNGGCWGCAG
805R primer: GACTACHVGGGTATCTAATCC

Multiplex_fwd primer:
AATGATACGGCGACCACCGAGA{TCTACAC}-[i5 index]-ACACTCTTTCCCTACACGACG
Multiplex_rev primer:
CAAGCAGAAGACGGCATACGAGAT-[i7 index]-GTGACTGGAGTTCAGACGTGTGCTCTTCCGATCT

PCR 1:

Denaturation	98C	30s		
Denaturation	98C	10s	\	
Hybridization	58C	30s	|	20X
Elongation	72C	6s	/	
Elongation	72C	2min		

PCR2:

Denaturation	98C	30s		
Denaturation	98C	10s	\	
Hybridization	58C	30s	|	15X
Elongation	72C	7s	/	
Elongation	72C	2min		

Reads were merged using:
SeqPrep version 1.1 (https://github.com/jstjohn/SeqPrep) John, J. St. "SeqPrep." (2011).
with default parameters.

SAMPLE	i7-index	i5-index
1A	AGGAGTCC	GTAAGGAG
2A	GCGATCTA	CTCTCTAT
2C1	GCGATCTA	AGAGTAGA
2C2	GCGATCTA	ACTGCATA
3A	GCGATCTA	AAGGAGTA
4A	ATAGAGAG	AGAGTAGA
8A	ATAGAGAG	AAGGAGTA
9A	TTCTGCCT	AGAGTAGA
11A	TTCTGCCT	ACTGCATA
11C1	GCTCAGGA	TAGATCGC
11C2	GCTCAGGA	TATCCTCT
12A	GCTCAGGA	GTAAGGAG
12C	GCTCAGGA	AAGGAGTA
13A	AGGAGTCC	CTCTCTAT
13C1	AGGAGTCC	ACTGCATA
13C2	AGGAGTCC	AAGGAGTA
16A	AGGAGTCC	CTAAGCCT
18A	CATGCCTA	GTAAGGAG
